# The Practice of Physical Activity After Breast Cancer Treatments: A Qualitative Study Among Portuguese Women

**DOI:** 10.3389/fpsyg.2022.823139

**Published:** 2022-03-15

**Authors:** Margarida Sequeira, Rita Luz, Maria-João Alvarez

**Affiliations:** ^1^CICPSI, Faculdade de Psicologia, Universidade de Lisboa, Lisbon, Portugal; ^2^CIIAS, Escola Superior de Saúde, Instituto Politécnico de Setúbal, Setúbal, Portugal

**Keywords:** female, cancer survivors, breast neoplasm, exercise, health behavior

## Abstract

**Background:**

Women survivors of breast cancer treatments face significant challenges around initiation or maintenance of physical activity (PA) as they transit to recovery. Embracing their needs and preferences is important to increase adherence. This study aimed to explore the perspectives of Portuguese women survivors of breast cancer regarding regular performance of PA and individual choices and strategies that should be considered in designing effective interventions.

**Methods:**

The individual semi-structured interviews (*N* = 20) were analyzed through thematic analysis, following an inductive process, seeking to identify the barriers, facilitators, and particular influencing circumstances associated with regular PA.

**Results:**

Four themes and several contextual, emotional, physical, and social factors were identified as determinants of regular PA. Guilt, women’s duties, and altruism to help close relatives were identified as emotional and cultural factors that are less-found in the existing literature. PA showed influence both from health professionals’ counseling and from knowledge and beliefs held by these women.

**Conclusion:**

While confirming the already-known impact of breast cancer diagnosis and treatments on PA, which redefine participants’ availability to be active, the findings highlight beliefs and specific emotional factors that should be considered when planning culturally sensitive interventions.

## Introduction

Cancer incidence is growing all over the world, reflecting the growth and aging of the population and the increase of cancer risk factors (Bray et al., 2018). Breast cancer has proven to be the leading cause of female death worldwide (Ferlay et al., 2015), accounting for almost one in four cancer cases among women, and similar numbers have been found in Portugal (Forjaz de Lacerda et al., 2018).

In most Western European countries, breast cancer mortality rates have decreased in recent years, especially in younger age groups, due to improvements in treatment and earlier detection (Senkus et al., 2015). The concept *breast cancer survivor* has emerged, defined by the US Centers for Disease Control and Prevention (CDC) as any person who has been diagnosed with breast cancer, a designation which will apply throughout their life (CDC, 2011). Three phases of survivorship have been defined by the American Cancer Society (2019): the *time from diagnosis to the end of initial treatment*, the *transition from treatment to extended survival*, and *long-term survival*. Breast cancer survivors are living longer and many live five or more years after diagnosis, under this designation of *long-term survivors* (Senkus et al., 2015; Alfano et al., 2019).

Benefits of regular physical activity (PA) and exercise after breast cancer diagnosis and treatments are demonstrated and include increased aerobic fitness, improved quality of life and activity tolerance, as well as reductions in fatigue, depressive symptoms, and therapeutic toxicity (Furmaniak et al., 2016; Lahart et al., 2018). Even so, a decrease in PA levels and quality of life has been reported as consequence of the effects associated with breast cancer diagnosis and its treatments (Bluethmann et al., 2015). PA is considered as any bodily movement produced by skeletal muscles that requires energy expenditure; *physical inactivity* is considered as an insufficient level of PA to meet present recommendations; exercise is considered as a subcategory of PA that is planned, structured, repetitive, and purposeful in the sense that the improvement or maintenance of one or more components of physical fitness is the objective (World Health Organisation, 2020).

The current recommendations for breast cancer survivors include avoiding inactivity and returning to normal daily activities as soon as possible after diagnosis, aiming for at least 150 min of moderate or 75 min of vigorous aerobic exercise per week and the inclusion of strengthening exercises at least 2 days per week (Campbell et al., 2019; Patel et al., 2019). These recommendations establish the minimum needed for someone to be considered an active person. Despite the long-known recommendations (Courneya et al., 2002) and the evidence of the health benefits of regular PA for people living with or beyond breast cancer (Patel et al., 2019), breast cancer survivors are not active enough to reach the benefits that PA can bring (Short et al., 2013). Nor have efforts to encourage and facilitate the regular practice of PA successfully integrated the routine of recovery and follow-up process for these patients in most centers (Stout et al., 2021). It is hence essential to better understand how to promote sustainable exercise behavior change in sedentary cancer survivors. The identification of the determinants for exercise behavior change among breast cancer survivors can help professionals to provide safe and effective PA recommendations for survivors and to target interventions toward women who find it more difficult to make positive changes after diagnosis (Anderson et al., 2017; Clifford et al., 2018).

An observational study developed in Portugal demonstrated that this population is interested in receiving counseling about exercise scheduling, programming, and advice regarding exercise practice (Ferreira et al., 2012). The authors identified the need to investigate possible associations between these cancer survivors’ preferences and the adherence and maintenance of these exercise programs, on the theory that better results are obtained when the preferences of patients are considered (Godinho et al., 2016; Bluethmann et al., 2017).

Environmental and cultural factors were also identified as limits in the choice of exercise setting (Schmitz et al., 2019). In a review, Clifford et al. (2018) identified that traditional family caregiving roles and lack of support from family were perceived as significant barriers to exercise among a cohort of specific cultural (Mexican American, Chinese American, and Korean American) groups of breast cancer survivors. This is not commonly reported among other studies and may provide some insight into cultural differences in attitudes toward exercise (Clifford et al., 2018). Additionally, the social value attached to PA can vary widely between cultures and change with time; for example, cycling may be perceived either as tiresome and socially undesirable, or it may become normative and even fashionable (Bauman et al., 2012).

Personal preferences and cultural and environmental factors must be considered when planning interventions to promote PA, and specific behavioral change techniques (BCT), delivered in different formats, may be considered for promoting PA among people after breast cancer; these BCTs should be identified based on the taxonomy developed by Michie et al. (2013) to describe the active components of health behavior change interventions.

This study aimed to explore the experiences and perspectives of Portuguese women survivors of breast cancer in their performance of PA, whether they were active in their practice or seeking to become active in the future. Specifically, we wanted to identify the factors that prevent these women from being active as well as the valued factors and strategies they could employ to accomplish the PA and exercise recommendations for breast cancer survivors—that is, the behavior change techniques/strategies they used spontaneously.

## Materials and Methods

### Participant Sampling

The sample was recruited by convenience from cancer patients’ associations and through a snowball sampling method, since women survivors of breast cancer were considered a hard-to-reach sample. Twenty adult Portuguese women in the survivorship phase of *transition from treatment to extended survival* (American Cancer Society, 2019), were contacted, and all agreed to participate. They were independent in their functioning and had no contraindication for performance of PA. Recruitment was performed by the people responsible by the patients associations and by the different volunteers, including a nurse.

### Study Design and Measures

This was a qualitative semi-structured interview study, supported by a script designed in advance (Table 1) and based on previous studies about PA and exercise among breast cancer survivors (Bauman et al., 2012; Ferreira et al., 2012; Sander et al., 2012; Hefferon et al., 2013; Kampshoff et al., 2014). We intended to examine in depth the individual perspectives and experiences of PA after breast cancer diagnosis and treatment—more specifically, to ascertain the patterns of PA practice and the beliefs, knowledge, reasons, and strategies spontaneously followed for regular PA.

**TABLE 1:** Interview script.

Discussion point/aims	Details
Introduction (not recorded)	Investigator introduced herself and presented the aims of the study, its potential benefits and the rights of the participants (specified below).
Participants’ sociodemographic and clinical data (not recorded)	Collected by the characterization questionnaire
PA practice: now, before, and after breast cancer	*What type of PA do you practice now?*
	*Before your breast cancer diagnosis, did you practice any physical activity?*
	*What did you do and how often?*
	*Has anything changed in PA after the breast cancer diagnosis?*
Determinants for regular practice of PA	*If you are active, what motivates you to be diligent in your physical activity?*
	*How do you feel when you practice?*
	*Do you feel any specific difficulties?*
	*If you are not active, why do not you do it?*
	*What difficulties do you feel or have, so that you are not active?*
Knowledge about advantages and recommendations about PA after Breast Cancer	*Do you know you can practice physical activity, even after breast cancer?*
	*Has this been told to you or recommended?*
	*Do you know physical activity has some advantages for breast cancer survivors?*
	*Do you know what and how much you can practice?*
Synthesis and meta-reflection about the interview.	*Do you have anything else you would like to add to this interview?*
	*What would you recommend to other BC survivors?*
Thank for participation.	*Thank you for your participation.*

*BC, Breast Cancer.*

The sample was recruited by convenience, from cancer patients’ associations and through a snowball sampling method, since women survivors of breast cancer were considered a hard-to-reach sample. Participants were contacted by the first author after informally agreeing to participate through the association or through other participants’ initial contact. The individual interviews were conducted in person by the first author between March and September 2019; they were performed in a location chosen by the participant and took from 20 to 60 min. The end of data collection was decided once the themes explored in the interviews seemed to be repeated and saturated.

As a first step, the investigator presented the aims of the study, its potential benefits and the rights of the participants. Participants were given the role of collaborators in the study, and it was explained that all the answers were important, with no possibility of answers being wrong. All the procedures were explained, including the use of a voice recorder, so the interviews could be heard, transcribed, and analyzed afterward. Data confidentiality was assured *via* the creation of a code for each participant: fictional name and the year of diagnosis. After this explanation and after obtaining the participant’s agreement, the informed consent was signed by both participant and investigator; only then was the voice recorder activated. A copy of the informed consent was sent to the participant by email.

The interview was complemented by a questionnaire that included sociodemographic (age, marital status, cohabitating family, residence district, level of education, professional status, activities outside work) and clinical data (date of the diagnosis, date and type of surgery, date of last surgery, oncoplastic and reconstructive surgery, adjuvant treatments). Data included in the questionnaire were previously identified as being related to regular PA practice among breast cancer survivors (Hefferon et al., 2013; Kampshoff et al., 2014). It was completed by the participant after the interview (in an online platform), and it was given the same code as the interview.

### Data Analysis

Upon completion of all the interviews, thematic analysis was led by the authors [MS] under an essentialists/realist paradigm, collecting experiences and identifying repeated patterns of meanings, following the six-stage process of thematic analysis described by Clarke and Braun (2013).

The interviews were performed in Portuguese and were transcribed verbatim, read and re-read by the first author in order to attain familiarity with the data before assigning relevant codes to passages of text. The transcripts were analyzed using an inductive, data-driven approach. The coding process was an analytic process and the first coding phase created codes that could capture both a semantic and conceptual reading of the data set (Clarke and Braun, 2013). This coding framework was developed in order to meet the aim of exploring the experiences and perspectives of Portuguese women breast cancer survivors regarding their performance of PA. Sub-themes were created to further specify aspects of the data. This coding phase ended by collating all the coded data relevant to each theme, aiming to summarize the main ideas and to check idiosyncrasies. When possible, in the interest of supporting the design of effective interventions in the future, behavior change techniques used spontaneously by participants in order to accomplish regular practice of PA were identified, based on the taxonomy defined by Michie et al. (2013).

The initial main themes and sub-themes were reviewed by the first and third authors, ensuring that the nature of each individual theme and the relationship between them would be clear (Clarke and Braun, 2013). Some themes were collapsed or regrouped. Themes were renamed, identifying the “essence” and constructing a concise, informative name for each, with both authors checking the attribution with 10% of the interviews. Inter-judge agreement was established with three other interviews (15%) following two steps: first, through unit analysis verification, then by computing the level of agreement [*k* = 0.726 (95% CI 0.57; 0.89), *p* < 0.0005]. In collaboration, both authors revised and created the final framework of themes and sub-themes, with minor discrepancies resolved *via* discussion. Data analysis was conducted using NVivo 12 software. The RATS checklist (Clarke and Braun, 2013) was followed to explicitly and comprehensively report the main characteristics of this qualitative study. Descriptive statistics was used to analyze sociodemographic and clinical data, using SPSS, version 25.

Informed consent and consent for publication were obtained from all individual participants included in the study. All procedures performed in the study were in accordance with the ethical standards of the institutional and/or national research committee and with the 1964 Helsinki declaration and its later amendments or comparable ethical standards. The study was approved by the Ethical Committee of the Faculty of Psychology of the University of Lisbon.

## Results

Participants were 20 women, breast cancer survivors in the transition from treatment to extended survival. Their ages were between 44 and 66 years old (*M* = 52, *SD* = 7.3); 16 were married, two single, and two divorced, two of them living alone. They had reached from 2 to 20 years post-diagnosis (*M* = 7, *SD* = 5.5). They all underwent surgery and at least one adjuvant treatment (chemotherapy, radiotherapy, hormonotherapy, or immunotherapy). Thirteen had graduated from university, and six had completed secondary school. Thirteen were currently working, two unemployed, four retired, and one on medical leave.

Regarding the regular practice of PA, nine out of 20 participants considered themselves active before diagnosis. However, three of these nine stopped regular PA afterward and six remained active. On the other hand, four of the non-active women before diagnosis started regular PA after. As a result, 10 out of the 20 participants considered themselves active by the time the interviews were performed.

The analysis of the interviews resulted in four key themes, independently of whether participants were regular with their PA practice. For each key theme we identified sub-themes that reflected more individualized narratives, all summarized in Table 2, where some citations were included. Barriers and facilitators are described together, since the sub-themes are similar. The chosen modalities of PA were also identified, showing the preferences of the participants, whether they were active or sought to become active.

**TABLE 2:** Key themes and sub-themes identified in the interviews.

Themes	Sub-themes	Examples:
Barriers to PA practice	Contextual	*The schedule for me was very important because weekends and the end of the day were not a choice. But, in the morning, I could not have teachers and classes available. (Celina2016)*
	Emotional	*I take pleasure from being in nature, but from the exercise, no, not pleasure from that. That discipline is not for me! (Kate2014)*
	Physical	*More tired, much more perspiration, it’s horrible! And also some joint pain. (Kate2014)*
	Social	*And when my friend is not coming, I would not either! (Celina2016)*
	Time unavailability	*My problem is the children’s schedule. Because with all the kids’ activities I do not have any available time at the end of the day, and I still have not managed to reconcile that. (Kate2014)*
Facilitators of PA practice	Contextual	*It was an excellent idea to open this type of gyms that do not have a rigid timetable. It’s a free regime—people go when they want or when they can. One can attend classes or not, can do individual things—machines or mats or cycling or rowing…(Chloe2016)*
	Emotional	*And it is good for your head, you do not think about it… you meet a person and see people passing by… you just get out of the house! (Emmy2016)*
	Physical	*And if we get fat, as happened with me—I started to increase weight and lose the ability to move. I could not go on like that. PA helps a person to become more active and to be in better shape and in a good mood. (Carol2014)*
	Social	*I happened to create a support group of friends and sometimes it was so hard to get out of bed that I appealed “I need someone to come to me, because I need to take a walk and I cannot get out of bed.” I did not feel like it, not physically nor psychologically. So there was always a friend who came to the door of the building and said “I’m already here waiting for you, take as long as you need, let’s go to the park!” and then we would go for a walk. Sometimes just with the phone—with the phones, it also works! (Celina2016)*
Information provided by health professionals	Advantages of regular practice of PA	*I really have to start because, besides the need to lose the weight I gained due to hormone therapy, I have my doctor’s pressure. He often insists, every time I meet him: “Stay thin, be happy, and do exercise.” (Meg2015)*
	Specific information about PA	*The surgeon said “so now you will start radiotherapy but that does not stop you from going out for a walk at the evening, I do not want you at home!” He said the ideal for me would be Pilates, yoga, things with low impact. I was always advised on that. (Hilary2015)*
	No specific information	*I happened to comment to my surgeon, as I go there every 6 months, that I used to run, and he just said: “yes very well.” (Els2010)*
	No recommendation	*No, no. Not by doctors. The conventional doctors I was followed by at the hospital, they did not even mention the subject. (Rose2013)*
Participants’ knowledge and beliefs about PA related to breast cancer	Improve social interaction and reduce isolation	*I think this type of lifestyle is a general recommendation for people who have cancer: walking, not standing still, not staying at home suffering… that I think is a settled point. (Kelly2016)*
	Prevent depression	*Because I think this is a prevention of depressive state. There are more difficult days in this process and walking prevents it. (Celina2016)*
	PA motivate more PA	*Because when we move, we also have a different reaction, our whole body reacts differently. (Carol2014)*
	Protection for cancer	*We have to do chemical treatment, right? Because if not, we are unable to quell the disease. But we also have to do our part—the other 50% we have to do ourselves. If we are not well with ourselves, if we are not feeling well, I think that everything becomes more complicated, more difficult. Even the way we look at the disease, right? And exercise is essential! (Bella2008)*

### Barriers and Facilitators

Most of the participants could identify barriers, and all participants identified at least one facilitator for regular PA, whether they were active on a regular basis or not. Time unavailability was identified as the most consistent barrier, and social support played an important role as a facilitator. We have identified contextual/extrinsic, emotional, physical, and social factors that have negative influence (barriers) or positive influence (facilitators) for regular PA practice.

Contextual factors such as unavailable sports facilities, difficult schedules and bad weather emerged as important limitations when one is trying to manage a regular physical activity, as mentioned:

*When the rain comes, often we all decide: “Ah, today it’s raining. Let’s do it tomorrow…” Yes, bad weather has an effect, mostly in winter. It’s better with sun!* (Els2010)

The same contextual factors such as good weather and having sports facilities nearby with flexible schedules were identified as a positive influence concerning the regular practice of PA, as indicated by several comments:

*If weather is good, we just get going!* (Sue2017)

Participants with a longer survivorship history also identified doctors’ attitudes and counseling as a contextual barrier, because those were discouraging or conflicting and stated:

*For us the problem is that one comes and says you cannot do yoga, another one says something else. We are pushed from one piece of information to another! That it’s a horrible thing so, then, comes a big intrinsic fear!* (Liz2001)

But for some others, with more recent diagnosis, the doctors’ and health professionals’ information and recommendations were an important facilitator to keeping an active lifestyle or, even, to becoming more active after diagnosis and treatments:

*(…) Quite soon after surgery, the doctor always advised me to walk by the sea shore, to do some little walks. No one ever told me not to do it.* (Kelly2016)

Intrinsic personal aspects related to low motivation for PA practice were identified as emotional barriers, and some examples were highlighted:

Lack of discipline and emotional unavailability, difficulty in achieving the appropriate rhythm or in resuming after an interruption:

*When I already had a regular practice, when it was no longer difficult to go and it even gave me some pleasure, I had to do a medical examination, and I had to stop for one month… (…) So, I was so excited at the time and it made me break the rhythm.* (Liz2001)

For some participants PA could become boring, beyond other reasons:

*Sometimes I did it on a regular basis, but ended up giving up. Sometimes because I did not have company, other times because it was boring and sometimes because I did not have time.* (Meg2015)

Surprisingly, our findings reminded us that wellbeing can also be identified as a possible barrier, because when someone feels good, it feels that one does not have to do anything different, and this fact can hinder PA, as one participant remarked:

*This difficulty for me is psychological, because I feel well now. If I had another problem or if I started feeling worse, I assure you, all my will would revive. This is very typical of human beings. I got used to getting up early to run or walk. I simply got into the habit. I have nothing to prevent me from doing it—it is an indolent attitude.* (Ivy1997)

Some participants mentioned feeling guilty for spending time on PA instead of on other family obligations:

*I find myself thinking that if I am not working I should spend more time with my daughter, or I should do some housework… I have all this in my mind. I have great difficulty in taking care of myself. I cannot do it, not yet!* (Hilary2015)

The emotional wellbeing promoted by the regular practice of PA was also mentioned as a facilitator. PA was mentioned as promoting strength and an active posture, as distracting from the worse symptoms and fears, as bringing energy and happiness, and as promoting self-esteem and the sensation of self-mastery, as indicated in several comments:

*Because it also helps us and I think that physical exercise transmits such great energy and happiness. Even if it was just half an hour, it was a need that made me feel good, willing to do things, cheerful—and, yes, I think that is fundamental.* (Bella2008)

The good mood and the psychological balance promoted by PA were also mentioned as positive emotional factors that could lead to regular practice of PA, as indicated here:

*It’s good for my mind—it’s not just good for the body, I think. I feel good, and perspiring a little is always good.* (Pauline2001)

*It is my psychological balance! The day-to-day at work is heavy, so I do it before going to work. That’s when I’m most reactive, that’s when I come to get my balance. Our self-image has changed; sometimes self-esteem decreases. It is a difficult period! And physical exercise ends up bringing our balance back.* (Suzi2004)

Most of the participants proved to have a positive proactive attitude toward their recovery, in particular concerning PA practice, even without specific or professional advice:

*I had the idea that I was about to die, but I also had the idea that I could not die. So I had to do my part, and that was my part, always trying to go beyond things, not complaining to anyone.* (Ivy1997)

Within positive emotional factors, some participants found that PA practice could be seen as a moment dedicated to themselves, allowing them to keep their regular lives and their social commitments, avoiding being the target of other people’s pity:

*“–So, what changed after the diagnosis (to make you willing to be more active)?”*

*“–I want to have more time for me, to have better quality of life!”* (Sue2017)

As another positive emotional factor, some participants showed curiosity, an emotionally and intellectually positive attitude towards knowledge about the disease and how to take control over it:

*Then I went to bookstores, I went everywhere to look for literature and there was very little, very little about it. But I found a book by chance. The author was a former doctor and he gave a good insight into cancer, how cancer develops, what we should do, the care that we should have, and I started to open up to other perspectives.* (Ivy1997)

Altruism and setting common goals with someone else were emotional factors identified as facilitators of regular PA. It was mainly the willingness to help others (husband) or not to demonstrate weakness that motivated PA:

*I believe that seeing my husband so sad gave me the strength to continue. I said, “No, I’m not going to show that I’m sad, I will show that I’m going to be stronger than this and I did that.” And believe me, I think that it made me be more active, you know? The fact that I saw him down made me go on and create weapons for myself.* (Carol2014)

Physical side effects of the treatments, pain, or other health problems were identified as a limitation to the regular practice of PA. Within this subject, participants clearly identified that fatigue or increased tiredness prevented or hindered this practice:

*I think I stopped because I could not do it anymore. It was a great physical tiredness. When I got home in the evening, I could not do it, I did not have the capacity to push myself anymore.* (Agatha2015)

At the same time, the physical wellbeing promoted by regular PA was also identified as a facilitator, and the importance of a good body image was mentioned as positively influencing PA practice, as stated by these two participants:

*Then, I discovered that I could go for walks, that I was not exposing my body. I could do the walking at my leisure time, with straps or without straps, with bras or prostheses, it did not matter, but I could do it at my will and nobody would notice my mastectomy.* (Mary2006)

The good sensations, meaning having positive feelings or reducing bad sensations during or after PA practice, were also mentioned within the physical wellbeing promoted by PA. Participants mentioned good sensations as:

Rejuvenating:

*Physically, I think I have rejuvenated. In that aspect I think I have rejuvenated—it was very good for me. All this movement makes me well!* (Anna2009)

Better sleep:

*One of the main things is because afterwards I sleep well. When I exercise, when I get tired, I sleep well. When not, I take long to fall asleep. So that’s one of the reasons why I like to do it.* (Emmy2016)

Good physical sensations and “addiction”:

*I feel good! And I’m quite sure that when I stop it…, I do not feel bad, but I can see the difference when I exercise and when I do not. I feel much better both psychologically and physically.* (Els2010)

Lack of social support was indicated as an important barrier to regular PA practice because, when alone, it was difficult to keep motivation and it was easier to find reasons not to do it:

*I used to run on my own, but it is more difficult, we always find ourselves an excuse not to go. But now, with the group I have their motivation. When they skip it, we all do… yesterday I did not go running because they did not go… so the company helps!* (Els2010)

However, when present, the social support, whether in general or coming specifically from family or friends, was seen as a positive influence for regular practice of PA, as noted by these participants:

*I go with my daughter, but it could be with a friend (…). If I do not feel like going, she says: “Ah no, today we go, we have to go!” I used to do it alone, but I gave up more easily—with my daughter it is easy to keep it up. In general, we go together and keep each other company.* (Pauline2001)

Difficulty with time management was the most mentioned barrier for regular practice of PA. Participants mentioned family obligations more often, but both family and work were preventing them from PA on a regular basis, as stated:

*I intend to practice in the near future. But first I have to take care of my old relatives. And as long as they depend on me, I would not have any time available.* (Mary2006)

*Work! Sometimes work stuff gets in the way and you cannot help it—then I would not go.* (Els2010)

In sum, we have identified contextual/extrinsic, emotional, physical, and social factors influencing regular practice of PA negatively (barriers) or positively (facilitators).

### Information Provided by Health Professionals

Health professionals’ information and recommendations appeared as important contextual barriers or facilitators in the decision to be active on a regular basis, as noted above. However, most of the participants mentioned having received different types of information from the health professionals they were followed by.

The most important information participants received from health professionals (doctors or others) included the advantages of regular practice of PA in the cancer survivorship process, for the control of symptoms and secondary effects, as these examples highlight:

*Yes. They mention it is a way to fight this type of disease—they mentioned it several times. I also had a psychology consultation and I remember the psychologist mentioned that exercise was important in our process, for people who had this type of problems, even for the future.* (Sue2017)

Some participants received specific recommendations concerning the type and duration of activity:

*He always said that I should do it. If I had difficulties, at least to do some walking or yoga… that was all mentioned. I am the one who really misbehaved! He told me, at least three times a week, water aerobics, walking, or yoga. And I should do it at least three times a week.* (Kate2014)

In other situations, health professionals were not specific on frequency, intensity, type, or duration of PA, but they agreed with participants’ initiatives and this agreement seemed to be important to these women, as one of them mentioned:

*“(…) Quite soon after surgery, the doctor always advised me to walk by the sea shore, to do some little walks. No one ever told me not to do it.”* (Kelly2016)

A few participants had no PA recommendations from the health professionals they had contact with, as answered by several:

*“–Following the treatments, did the doctor ever advise or discourage doing physical activity?”*

*“–I never asked, but I was never told.”* (Anna2009)

Indeed, participants mentioned actual health professionals’ information, recommendations, and agreement as an important positive influence concerning PA. However, only some of them got a general recommendation to be active. Very few recommendations were specific on the type of activity, and none of the participants received concrete information about frequency, intensity, and duration of PA practice, in terms of the international recommendations—neither from doctors nor from other health professionals.

### Knowledge and Beliefs

Knowledge and beliefs about PA practice or specific lifestyles after breast cancer were mentioned by half of the participants, coming either from health professionals, common sense, or other sources.

Some believed that social interaction was important and that isolation could be a problem in breast cancer recovery, so PA could help improve social interaction and reduce isolation, as they mentioned:

*I also think it is assumed, at least for me, that despite having cancer, one should not stay at home and should not to be pushed down by the disease or the situation. So, yes, we should do it: walking, going out to the beach… With moderation, because of the sun and fatigue, but yes, I think so.* (Chloe2016)

Some participants agreed that PA would prevent a depressive state after diagnosis and treatment:

*Because I think this is a prevention of depressive state. There are more difficult days in this process and walking prevents it.* (Celina2016)

Some others believed that regular practice of PA could motivate more PA, because they believe movement generates more movement and action generates more action.

Finally, some participants believed that regular practice of PA could protect them from recurrence and that it plays an active role in the recovery/survivorship process, as they state:

*In relation to breast cancer, what I heard is that those who had cancer and practice sport are trying to protect themselves, so that does not happen again. I do not know if the studies are reliable or not, but there are people who say that stress levels and lack of physical exercise are two very important factors for the development of cancer. I believe it is so!* (Chloe2016)

The knowledge and beliefs about PA practice for breast cancer survivorship that these survivors hold and mentioned were mainly related to the advantages of PA for this process and were identified as positively influencing their decision to follow an active, healthy lifestyle.

There were also some beliefs around what concerns the type of PA activity to be practiced. It was stated that people should look for the PA type that “speaks to us,” to discover an activity that one enjoys and that would bring some pleasure (Rose2013). Some believe that running was not recommended and that they should mainly be focused on walking, preferably outdoors, not in shopping centers (Sue2017). Most of the participants mentioned walking and hiking as the best thing they could do.

Indeed, running was the least frequent PA type within this group, practiced only by one of the active participants. As mentioned, some did not recommend it at all. Walking seemed to be the more frequent choice, allowing participants to be free with their schedules and time management. Gymnastics was identified as motivational activity, as it is a group activity. Yoga, Pilates, hydro-gymnastics, and swimming were also chosen by some participants as no-impact and lower-intensity activities, following some health professionals’ recommendations.

## Discussion

This study extended previous research done with Portuguese women survivors of breast cancer (Ferreira et al., 2012) by examining specific determinants for regular practice of PA. Although resource-intensive, the qualitative nature of this study allowed a comprehensive understanding of participants’ subjective perspectives on barriers and facilitators, the role of health professionals, knowledge and beliefs, and choices regarding regular practice of PA, not often attainable through quantifiable measures (Clifford et al., 2018). This study indicates that regular PA for women after breast cancer is a difficult behavior to address due to its complexity and the multilevel influencing factors.

### Positive and Negative Influences for Physical Activity

Most of the barriers identified are consistent with previous studies, whether they include cancer survivors or other chronic patients (Sander et al., 2012; Clifford et al., 2018), including sports facilities being unavailable, bad weather, fatigue, or lack of social support. Once again, time management emerges as an important barrier to regular PA for these women, proving to be a difficult barrier to address. Nevertheless, some of our participants demonstrated a positive proactive attitude towards the practice of PA, allowing them to be active and to overcome time constraints, which has not been commonly reported among other studies (e.g., Bauman et al., 2012; Clarke and Braun, 2013). Participants mentioned that seeking to help others, finding quality time for themselves or the emotional wellbeing brought by PA practice were motivations that help them to overcome time management difficulties and allow them to be more active.

This analyses highlight some emotional factors not identified in previous studies with breast cancer survivors (e.g., Courneya, 2009; Bauman et al., 2012). These include the guilt from spending time on exercise activities instead of being with family and the large weight of family commitments being identified as great barriers by several participants with younger children or elderly to take care of. In contrast, the responsibility and altruism to help close relatives accomplish their own regular PA was indicated as a facilitator. These specific factors seem to be closely related to cultural aspects of a society that relies on women to keep the home and family functioning (Wall et al., 2016). This was also evidenced in the unavailability of time for regular PA largely being related to family commitments. Interventions seeking to promote PA among Portuguese women after breast cancer should therefore include planning strategies. Physical limitations such as treatment side effects or joint pain were identified as barriers to exercise, but fatigue seems to be the greatest physical limitation, this being mentioned by all the participants who identified physical barriers. This is the main symptom for the majority of survivors of cancer, being present in 70% of all cancer survivors after chemo or radiotherapy, and it very often prevents regular practice of PA (Clifford et al., 2018). Physical well-being was in turn identified as a facilitator and motivator for a regular PA practice, bringing a sensation of rejuvenation, promoting better sleep quality, facilitating more good physical sensations, and “addiction” to being regularly active. The attraction of these good sensations was widely evidenced on the part of both active and not-regularly active participants, indicating a possible motivation for the use of these ideas in future interventions. This direct relation of the good sensations to a more active lifestyle is reinforced in several studies that relate regular PA with a consequent increase in the quality of life in the physical, emotional, social, intellectual, or mental domains (Lahart et al., 2018; Patel et al., 2019). Trying to keep or restore a good body image is also a relevant physical facilitator for PA for some of our participants. It was also mentioned by breast cancer survivors in another qualitative study (Sander et al., 2012), and it relates to an important recommendation on weight control, which is often difficult for these women to accomplish, especially during and after chemotherapy (Kirkham et al., 2016).

Social support from family and friends had a central role and was considered mandatory in the early stages of the disease. It appears as a motivation to get out of bed on the most difficult days or to accomplish the daily goals of doing some walking. Family is the most present and available support, but groups of friends were built up specifically after diagnosis and treatment. Indeed, social support had been previously identified as being of great influence in the regularity of PA practice, but this was mainly regarding long-term maintenance of PA practice (Emery et al., 2009; Sander et al., 2012), whereas we found its importance mainly in the early stages after treatment. When social support is perceived as low or absent, participants feel less pleasant about the PA practice, leading them to give up, even among participants who have set specific goals and established long-term regular practice of PA. It seems clear that social support is an important facilitator to be kept under consideration in future interventions and should be considered from the early stages of the disease. Additionally, survivors without direct support systems such as family or friends should be encouraged to link up with groups in their neighborhoods through social media or by direct contact, in view of importance that has been identified for social support.

### The Perceived Role of Health Professionals

Health professionals have a vital role in changing health behaviors, and their actions have been identified as important to the final decision to engage in an active lifestyle and to feeling safe in that practice (WHO, 2016). Historically, after breast cancer, women were advised to avoid or limit exercise—specifically resistance exercise—because of the risk of developing lymphedema or other complications (Sander et al., 2012). This fact was clearly evidenced by two participants with a longer survivorship history, who got discouraging or at least conflicting messages about PA from their doctors. Participants with more recent diagnoses mentioned they were generically advised to be active and some modalities and advantages were mentioned, but none of them got specific information on duration and intensity of exercise from health professionals, despite these recommendations for breast cancer survivors being well-known and long-delivered (Courneya et al., 2002). Even so, some participants showed a positive attitude toward being regular with their PA and they sought approval from their medical doctors. This positive attitude and spontaneous behavior change after breast cancer diagnosis and treatment were identified by Anderson et al. (2017) but concerning diet changes and alcohol intake reduction, being less evident for regular PA practice.

Some participants received recommendations from their medical doctors to be active and to change some other life habits, but despite their doctors’ important influence, some women could not muster an active attitude, mentioning low motivation, lack of discipline and rhythm, or emotional unavailability to accomplish the few recommendations. Some of these barriers were previously mentioned when studying long-term (more than 5 years) determinants of PA practice (Emery et al., 2009; Hefferon et al., 2013). Additionally, it is known that behavior change is difficult to achieve, so the type of information and recommendations provided by health professionals should be patient-tailored, responding to specific needs and doubts. From this study, we found that giving general recommendations for PA may be insufficient to induce a regular practice among this group of women. The competency of the person delivering the information and the quality of behavior change interventions will, in part, determine their effectiveness and the adherence to the recommendations (Dixon and Johnston, 2021). Some mismatch between the needs, the information, and the way it was delivered could therefore explain the non-compliance of some participants.

The lack of specific information provided by health professionals was identified and must be addressed; there is an evident need to change messages surrounding exercise type and intensity that is safe and effective, as survivors often mention they “don’t know what to do” (Clifford et al., 2018). Participants sought specific information in the available literature, in a positive, active attitude about changing their lifestyle after breast cancer diagnosis. Some others mentioned they believed that an active lifestyle was important to prevent isolation and depression, to help with the course of the disease and survivorship, and that each cancer survivor has an important role to play in that. Unlike Costanzo et al. (2011), who found no relationship between the belief that exercise could prevent a recurrence and an increase in PA, such beliefs induced some of our participants into being regularly active.

Health and governmental sectors have a direct influence on the regular practice of PA of breast cancer survivors (Bauman et al., 2012), and health professionals play an important role when providing well-informed, tailored, and well-delivered recommendations on their regular practice of PA (Dixon and Johnston, 2021). This role can be enhanced by the availability of facilities and flexible working hours that are important contextual determinants of PA practice. Knowledge of the behavioral change strategies that influence the practice of PA among Portuguese women survivors of breast cancer will allow health professionals to adjust their messages to the specific cultural context in order to facilitate adherence to the recommendations (Anderson et al., 2017; Clifford et al., 2018; Patel et al., 2019). Health professionals should therefore be trained to understand the importance of PA and to include a discussion about this when caring for breast cancer patients.

### Beliefs, Preferences, Cultural Context, and Tailored Interventions

A large portion of both active and less-active participants chose walking as the preferred type of exercise, mainly at a moderate intensity, beginning either immediately or longer after completing treatment, as also found by other studies with breast cancer survivors (Anderson et al., 2017; Clifford et al., 2018). This suggests that efforts to determine specific barriers to aerobic or resistance exercise must help identify strategies for increasing participation in higher-intensity PA in this population, considering the more recent recommendations (Campbell et al., 2019; Patel et al., 2019).

While providing insights on the determinants of PA practice that are consistent with previous studies and samples, this study brings further knowledge on two important aspects. First, regular PA among these Portuguese women is influenced by culturally determined factors, such as the central role of women in the functioning of the home/family and the importance of social support, which must be considered when planning an intervention seeking PA promotion (Bluethmann et al., 2017). Second, the women spontaneously use specific strategies that help them to engage in regular PA practice and, due to their ecological validity, these should be considered in future interventions.

Through this study, a group of personal strategies that are perceived by these Portuguese women to be effective for engaging in regular PA was identified; this makes available some behavior change techniques that could be effective in helping overcome barriers and in making use of the identified strategies and facilitators for promoting regular PA in this population. Tailored interventions may hence result in increased practice of PA when specific behavior change techniques are applied (Bluethmann et al., 2017), those spontaneously used by breast cancer survivors. Using the BCT Taxonomy described by Michie et al. (2013), we can identify in our results some of these techniques according to the groups focused on: goals and planning (Group 1—goal setting and commitment); feedback and monitoring (Group 2—self-monitoring of outcomes of behavior); social support (Group 3); techniques focused on shaping knowledge (Group 4—behavioral experiments); natural consequences (Group 5—information about health consequences, monitoring of emotional consequences; information about natural consequences); comparison of outcomes (Group 9—credible source); regulation (Group 11—reducing negative emotions, conserving mental resources); identity (Group 13—valued self-identity); and self-belief (Group 15—verbal persuasion about capability and self-talk).

The identification of the specific views, needs, and preferences of Portuguese women after breast cancer highlighted in this study is fundamental for informing future interventions to promote regular practice of PA (Alfano et al., 2019) among this group of women.

### Limitations

This study is not without limitations. The determinants that we found important to address in tailored interventions in the future may be too specific to a particular cultural context, and the most idiosyncratic determinants may not be relevant to other samples. Although we found saturation of themes in the interviews, there were still only 20 participants, so we must consider the possibility that we would find different results with other types of qualitative methods, such as field observations. Reducing distortion involves raising awareness of the research objectives and confidentiality, which was ensured. However, despite the advantages of individual face-to-face interviews—such as more flexibility in collecting information and greater connection to the interviewer and the study, when compared to other methods (e.g., questionnaires or online interviews)—they are permeable to social desirability, and participants may have displayed proactive advantages and attitudes resulting more from this image management than from what they actually think about PA after breast cancer.

## Conclusion

The study helped to identify barriers as well as strategies that Portuguese women spontaneously put into practice to facilitate engagement in regular PA after breast cancer. This information gives us clues for behavior change techniques that could be effective in helping overcome barriers and in promoting regular PA among this specific group of women, considering the specifically tailored interventions that can be developed in conjunction with the currently known international recommendation for practice of PA for breast cancer survivors. Less-frequently featured in previous literature, our study identified the importance of emotional barriers that may result from cultural specificities, such as family commitments and guilt over spending time on exercise activities. In the same vein, facilitators included the altruism to help others with their regular PA, the individual’s positive attitude toward being active, the belief that an active lifestyle could be important for the good course of the disease and survivorship, and that each cancer survivor has an important role to play in this. This adds different determinants that may be specific to this sample or might be relevant in some other societies that value collectivism, through the emphasis on the social role of women. Because of the particular cultural context and some idiosyncratic determinants, the factors identified in this study are relevant to future interventions in similar contexts.

## Data Availability Statement

The raw data supporting the conclusions of this article will be made available by the authors, without undue reservation.

## Ethics Statement

The studies involving human participants were reviewed and approved by Comissão de Ética da Faculdade de Psicologia da Universidade de Lisboa (Ethical Committee of the Faculty of Psychology of the University of Lisbon). The patients/participants provided their written informed consent to participate in this study.

## Author Contributions

MS and M-JA contributed to conception and design of the study and performed data analysis. MS performed data collection, material preparation, and wrote the first draft of the manuscript. All authors commented on previous versions of the manuscript, revising it for intellectual content, and read and approved the final manuscript.

## Conflict of Interest

The authors declare that the research was conducted in the absence of any commercial or financial relationships that could be construed as a potential conflict of interest.

## Publisher’s Note

All claims expressed in this article are solely those of the authors and do not necessarily represent those of their affiliated organizations, or those of the publisher, the editors and the reviewers. Any product that may be evaluated in this article, or claim that may be made by its manufacturer, is not guaranteed or endorsed by the publisher.
